# Modeling across-trial variability in the Wald drift rate parameter

**DOI:** 10.3758/s13428-020-01448-7

**Published:** 2020-09-18

**Authors:** Helen Steingroever, Dominik Wabersich, Eric-Jan Wagenmakers

**Affiliations:** 1 Department of Psychology, University of Amsterdam, PO Box 15906, Amsterdam, 1001 NK The Netherlands; 2 Department of Psychology, University of Tübingen, Tübingen, Germany

**Keywords:** Cognitive modeling, Evidence accumulation, One-choice decision tasks, Reaction time modeling, Decision-making, Inverse Gaussian distribution

## Abstract

The shifted-Wald model is a popular analysis tool for one-choice reaction-time tasks. In its simplest version, the shifted-Wald model assumes a constant trial-independent drift rate parameter. However, the presence of endogenous processes—fluctuation in attention and motivation, fatigue and boredom—suggest that drift rate might vary across experimental trials. Here we show how across-trial variability in drift rate can be accounted for by assuming a trial-specific drift rate parameter that is governed by a positive-valued distribution. We consider two candidate distributions: the truncated normal distribution and the gamma distribution. For the resulting distributions of first-arrival times, we derive analytical and sampling-based solutions, and implement the models in a Bayesian framework. Recovery studies and an application to a data set comprised of 1469 participants suggest that (1) both mixture distributions yield similar results; (2) all model parameters can be recovered accurately except for the drift variance parameter; (3) despite poor recovery, the presence of the drift variance parameter facilitates accurate recovery of the remaining parameters; (4) shift, threshold, and drift mean parameters are correlated.

Human decision-making has been studied using a large variety of experimental paradigms. One of the most elementary tasks requires that participants respond immediately after detecting the onset of a stimulus. The key dependent variable in these tasks is reaction time (RT), the time from stimulus onset to participants’ execution of the motor response (usually a key press). Examples of such tasks include simple RT tasks (chapter 2 in Luce, 1986; Smith, 2000), go/no-go tasks (Heathcote, 2004; Schwarz, 2001), temporal-cueing tasks (Jepma et al., 2012), the psychomotor vigilance test (Ratcliff & Van Dongen, 2011), the brightness detection task (Ratcliff & Van Dongen, 2011), the braking task (Ratcliff & Strayer, 2014), and the driving-around task (Ratcliff & Strayer, 2014).

Data from these RT tasks can be analyzed with the shifted-Wald model (SW; Fig. 1). The SW model is based on the Wald distribution (Wald, 1947; also known as the inverse Gaussian distribution) which represents the density of the first-arrival times of a Wiener diffusion process toward a single absorbing boundary. The basic model has three parameters that correspond closely to the three parameters of the Ratcliff diffusion model (Ratcliff, 1978; Forstmann et al., 2016; Ratcliff et al., 2016): (1) the decision threshold *α*, that is, the distance from the starting point (which we arbitrarily set at 0) to the absorbing barrier; (2) the drift rate *ξ* of the diffusion process; and (3) the shift parameter *𝜃* that quantifies the time required for nondecision processes (see below for a more detailed description of the model).

**Fig. 1:**
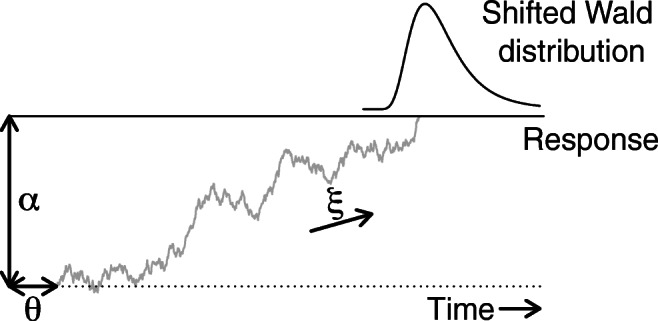
An illustration of the shifted-Wald model. The model parameters are the decision threshold *α*, the shift parameter *𝜃*, and the drift rate *ξ*. In its simplest version, the shifted-Wald model uses a constant drift parameter. However, in the implementations that we describe below we use a trial-dependent drift parameter that follows either a truncated normal distribution or a gamma distribution

The vanilla SW model assumes a constant drift rate across trials. However, the presence of endogenous processes, such as fluctuation in attention and motivation, fatigue and boredom, suggest that drift rate might vary across trials (Ratcliff & Strayer, 2014; Ratcliff & Tuerlinckx, 2002; Ratcliff & Van Dongen, 2011; Ratcliff & Van Zandt, 1999): “Parameters may also change from day to day or from one block of trials to the next. Evidence of such drift can be found in the variability of block mean reaction times” (p. 121; Burbeck & Luce, 1982). In order to incorporate across-trial variability in the Wald drift rate parameter *ξ*, the standard approach is to assume that its value on any specific trial is governed by a certain positive-valued distribution *h* with parameters ***γ***, that is, $\xi \sim h (\boldsymbol {\gamma })$. Subsequently, in order to derive the distribution of the first-arrival times *t* assuming a trial-dependent drift parameter, one has to integrate out drift rate, that is, $f (t ; \alpha , \theta , \boldsymbol {\gamma }) = {\int \limits }_{0}^{\infty } \text {SW}(t ; \alpha , \ \theta , \ \xi ) \cdot h(\xi ; \boldsymbol {\gamma } ) \mathrm {d}{\xi }$.

How to choose an appropriate distribution *h*(*ξ*;***γ***) for the drift rate parameter? Ideally, such a distribution should be (a) plausible (e.g., allowing only positive drift rates); and (b) allow for a closed-form expression *f*(*t*;*α*, *𝜃*, ***γ***). Here we explore two candidate distributions for *h*(*ξ*;***γ***): the truncated normal distribution and the gamma distribution. This general approach to incorporate across-trial variability in the Wald drift rate parameter has been explored in earlier work. Specifically, Desmond and Yang (2011) showed that a closed-form expression for *f*(*t*;*α*, *𝜃*, ***γ***) exists when drift rate follows a truncated normal distribution, and when the SW model has decision threshold *α* fixed to one and shift parameter *𝜃* fixed to zero (see Weiss, 2012, for an application). This scenario deviates from ours since we want threshold and shift parameters to be free, but the general approach is the same (see Whitmore, 1986, for a scenario that deviates even more from ours, but that employs the same general approach). Logan et al., (2014) also use this general approach, but their goal was to incorporate across-trial variability in the threshold parameter.

In this article, we derive the distribution of the first-arrival times for the SW model assuming a trial-dependent drift rate parameter. We also explore an alternative, sampling-based approach. We present Bayesian implementations of both approaches. The adequacy of these implementations is confirmed in a series of parameter recovery studies. Finally, the practicality of our methodology is demonstrated by fitting the extended SW models to a data set comprised of 1469 participants (Woods et al., 2015).

## The shifted-Wald model

The shifted-Wald model gives the density of the first-arrival times of a Wiener diffusion process toward a single absorbing boundary. Figure 1 shows how the model conceptualizes the decision-making process as a single-boundary diffusion process. In particular, the model assumes that evidence is accumulated with a drift rate *ξ* until an absorbing barrier *α* is reached. Additional delay time is captured by *𝜃*—the time required for nondecision processes.

The density of the first-arrival times is known as the Wald distribution or the inverse Gaussian distribution (Anders et al., in press; Donkin et al., 2009; Wald, 1947), and it is given by:

1 $$ \begin{array}{@{}rcl@{}} \text{SW}(t ; \ \alpha, \ \theta, \ \xi) &=& \frac{\alpha }{\sqrt{2\pi (t - \theta)^{3}}} \exp\\ &&\times \left( - \frac{[\alpha - \xi (t - \theta)]^{2}}{2(t - \theta)} \right),  t,  \alpha,  \xi \geq 0,  t > \theta, \theta \geq 0 \end{array} $$

with *t* the first-arrival time. The Wald distribution has a positively skewed unimodal shape as shown in the top of Fig. 1. The extension of the SW model that we consider here assumes across-trial variability in the Wald drift rate parameter either according to a truncated normal distribution or a gamma distribution.

## Across-trial variability in the Wald drift rate parameter

Across-trial variability in Wald drift rate parameter *ξ* can be incorporated by assuming that drift rate follows a positive-valued distribution, that is, $\xi \sim h (\boldsymbol {\gamma })$. Two candidate distributions for *h*(*ξ*; ***γ***) are the truncated normal distribution and the gamma distribution. In order to implement the resulting distribution of the first-arrival times, we use two different approaches. Firstly, we derive the analytical solution for the distribution of the first-arrival times by integrating out drift rate, that is, $f (t ; \ \alpha , \theta , \boldsymbol {\gamma }) = {\int \limits }_{0}^{\infty } \text {SW}(t ; \ \alpha , \ \theta , \ \xi ) \cdot h(\xi ; \ \boldsymbol {\gamma }) \mathrm {d}{\xi }$. Secondly, we explain a sampling-based implementation, that is, on each trial, we assume that RT follows a SW distribution that depends in part on a trial-dependent drift rate that is itself a draw from a positive-valued distribution.

### SW-TN mixture

The first candidate distribution for across-trial variability in the Wald drift rate parameter *ξ* is the truncated normal (TN) distribution. The TN distribution is a normal distribution that can be bounded below and above (Robert, 1995). Since we are interested in a positive-valued distribution on drift rate, we consider here a TN distribution that is bounded below by zero and unbounded above. The probability density function of the drift rate *ξ* is then given by:

2 $$  \text{TN}(\xi; \ \mu_{\xi}, \ \sigma_{\xi}, \ a=0, \ b=\infty) = \left \{\begin{array}{l l} \frac{\frac{1}{\sigma_{\xi}} \phi \left ({\frac{\xi - \mu_{\xi}}{\sigma_{\xi}}} \right )}{ {\Phi}{\left (\frac{ \mu_{\xi}}{\sigma_{\xi}} \right )}} & \text{for}  \xi \geq 0 \\ 0 & \text{elsewhere,} \end{array} \right. $$

with $\mu _{\xi } \in \mathbb {R}$ and *σ*_*ξ*_ > 0. The free parameters are the location parameter $\mu _{\xi } \in \mathbb {R}$, and the scale parameter $\sigma _{\xi } \in \mathbb {R}^{+}$. Parameters *a* and *b* are the lower and upper bounds of the TN distribution, respectively, and *ϕ*(⋅) is the probability density function of the standard normal distribution. Thus, $\phi (x) = \frac {1}{\sqrt {2 \pi } } \exp \left (-\frac {1}{2} x^{2} \right ) $, and in particular, $\phi \left ({\frac {\xi - \mu _{\xi }}{ \sigma _{\xi } }} \right ) = \frac {1}{\sqrt {2 \pi } } \exp \left (-\frac {(\xi - \mu _{\xi } )^{2}}{2 \sigma ^{2}_{\xi }} \right ) $. Finally, Φ(⋅) is the cumulative distribution function of the standard normal distribution.

The analytical solution for the distribution of the first-arrival times can be derived by “completing the square” and some algebraic manipulations. The result is what we will term the SW-TN mixture:^1^

3 $$  \begin{array}{ll} f(t; \ \alpha, \ \theta, \ \mu_{\xi}, \ \sigma_{\xi}) &= {\int}_{0}^{\infty} \text{SW}(t ; \ \alpha, \ \theta, \ \xi) \cdot \text{TN}(\xi; \ \mu_{\xi}, \ \sigma_{\xi}, \ a=0, \ b=\infty) \mathrm{d}{\xi} \\ &= {\int}_{0}^{\infty} \frac{\alpha}{\sqrt{2\pi (t - \theta)^{3}}} \cdot \exp \left( \frac{- [\alpha - \xi (t - \theta)]^{2}}{2 (t - \theta)} \right) \cdot \frac{\frac{1}{\sigma_{\xi}} \ \phi{ \left (\frac{\xi - \mu_{\xi}}{\sigma_{\xi}} \right )} }{\Phi{ \left (\frac{ \mu_{\xi}}{\sigma_{\xi}} \right ) }} \ \mathrm{d}{\xi} \\ &= \frac{\alpha} {\sqrt{2 \pi (t - \theta)^{3} \left [ (t - \theta) \sigma^{2}_{\xi} + 1 \right ]} } \cdot \frac{1}{\Phi{\left (\frac{\mu_{\xi}}{\sigma_{\xi}} \right )}} \cdot \\ & \ \ \ \ \exp \left (- \frac{ \left [ \mu_{\xi} (t - \theta) - \alpha \right ]^{2}} {2 (t - \theta) \left [ (t - \theta) \sigma^{2}_{\xi} + 1 \right ]} \right) \cdot {\Phi} \left (\frac{ \alpha \sigma^{2}_{\xi} + \mu_{\xi}}{\sqrt{ \sigma^{2}_{\xi} \left [ (t - \theta) \sigma^{2}_{\xi} + 1 \right ]} } \right ). \end{array} $$

To the best of our knowledge, the distribution given in Eq. 3 has not been derived before; however, the method for obtaining the result followed the approach by Desmond and Yang (2011; see also Logan et al., 2014; and Whitmore, 1986). In contrast to Desmond and Yang, we let the decision threshold and shift parameter free to vary.

### SW-GAM mixture

A disadvantage of the TN distribution is that it restricts the values of drift rate *ξ* to a specified interval [*a*, *b*] artificially and abruptly. An alternative candidate distribution that is naturally restricted to positive values is the gamma distribution. For this reason, it has been argued that the gamma distribution is conceptually more attractive (Terry et al., 2015). The probability density function of the gamma distribution parameterized by the shape parameter $\kappa \in \mathbb {R}^{>0}$ and the rate parameter $\tau \in \mathbb {R}^{>0}$ is given by:

4 $$  \text{GAM}(\xi; \ \text{shape} = \kappa, \ \text{rate} = \tau) = \left \{\begin{array}{l l} \cfrac{{\tau^{\kappa}} \ \xi^{\kappa - 1} \ \exp(- \tau \xi )}{ {\Gamma}({\kappa)}} & \text{for}  \xi \geq 0 \\ 0 & \text{elsewhere,} \end{array} \right. $$

with *κ* > 0, *𝜃* > 0 and where Γ(*κ*) is the gamma function evaluated at *κ*.

Analogous to the SW-TN mixture, we assume that RT follows a SW distribution with a drift rate parameter that varies across trials according to a gamma distribution. The next step is to integrate out drift rate resulting in the analytical solution of $f (t ; \ \alpha , \ \theta , \ \kappa , \ \tau ) = {\int \limits }_{0}^{\infty } \text {SW}(t ; \ \alpha , \ \theta , \ \xi ) \cdot \text {GAM}(\xi ; \ \kappa , \ \tau ) \mathrm {d}{\xi }$. To obtain this solution, we tried standard integration techniques, and also used the computer software Maple and Mathematica (Maple, 2015; Wolfram Research, 2010). The main analytical solution is displayed in Fig. 12 of the appendix.^2^ It is evident that the analytical solution is extremely complicated, that is, lengthy and containing the Laguerre polynomial (chapter 3 in Bayin, 2006; Koepf, 1997; more details can be found in the appendix).

For the practical implementation of the analytical solution, we could use the lengthy equation shown in Fig. 12. However, it appears more insightful and efficient to use a for-loop implementation in a probabilistic programming language. This means that on each trial, we assume that RT is a draw from a SW distribution that depends in part on a trial-dependent drift rate parameter—a parameter that is itself a draw from a gamma distribution with shape parameter *κ* and rate parameter *τ*. We refer to this solution as the SW-GAM mixture.

Note that the mean of the gamma distribution is given by *μ*_*ξ*_ = *κ*/*τ* and the variance by $\sigma ^{2}_{\xi } = \kappa / \tau ^{2}$. This is equivalent to $\kappa = \mu _{\xi }^{2} / \sigma _{\xi }^{2}$ and $\tau = \mu _{\xi } / \sigma ^{2}_{\xi }$. The advantage of expressing the parameters of the gamma distribution in terms of the mean and the variance is that this allows us to easier compare results of the SW-TN and SW-GAM mixture.

### Bayesian implementation of the two mixtures

In order to be able to apply the models to data and draw inferences about their parameters, we implemented the SW-TN and SW-GAM mixtures in a Bayesian framework using the software JAGS (Plummer, 2003; for related Bayesian work see Banerjee & Bhattacharyya, 1979; Betro & Rotondi, 1991). In the case of the SW-TN mixture, we added the analytical solution (Eq. 3) to JAGS as described in the tutorial by Wabersich and Vandekerckhove (2013). For the implementation of the SW-GAM mixture, we added the constant-drift SW distribution (Eq. 1) to JAGS—again following the tutorial by Wabersich and Vandekerckhove (2013)—and then used a for-loop construction (the model file is presented in the Appendix). For both mixtures, the prior distributions for the model parameters were inspired by Jepma et al., (2012; second experiment). These prior distributions are uninformative within a range that is plausible for data from one-choice RT tasks. In particular, we chose the following prior distributions:

- $\alpha \sim \mathcal {N}^{+}(\mu = 2, \sigma = \sqrt {\frac {5}{2}} )$
- $\theta \sim \mathcal {N}^{+}(\mu = .180, \sigma = \frac {1}{2} )$
- $\mu _{\xi } \sim \mathcal {N}^{+}(\mu = 8, \sigma = \sqrt {10} )$
- $\sigma ^{2}_{\xi } \sim \mathcal {U}(0, 5)$.

For all model applications reported in this article, we used random starting values, and simultaneously ran three Markov chain Monte Carlo (MCMC) chains. To assess whether the MCMC chains of all parameters had converged successfully from their starting values to their stationary distributions, we visually inspected the chains. In addition, we used the $\hat {R}$ statistic (Gelman & Rubin, 1992), a formal diagnostic measure of convergence that compares the between-chain variability to the within-chain variability. As a rule of thumb, values of $\hat {R}$ close to 1.0 indicate adequate convergence of the chains from their starting values to their stationary distributions, whereas values greater than 1.1 indicate inadequate convergence. All relevant code is available on GitHub (https://github.com/HelenSteingroever/jags-wald/releases).

## Recovery studies

### Methods

Before fitting the SW-TN and SW-GAM mixtures to real data, it is important to confirm that the two models can accurately recover parameters (Heathcote et al., 2015). An informative parameter recovery study uses a representative number of synthetic participants, a representative number of trials, and representative parameter values. We obtained these representative values by considering the performance of 1469 participants who each contributed 120 trials (Woods et al., 2015; see below for more details on the data set). In our simulation study, we use two generated data sets, one containing the—what we will call in the remainder—SW-TN synthetic participants, and the other containing the SW-GAM synthetic participants. We generated 30 synthetic SW-TN participants and 30 synthetic SW-GAM participants each contributing 120 trials (as in Woods et al., 2015). These synthetic participants were generated with representative parameter values that were obtained as follows: First, we selected 30 participants from Woods’ data set who spanned a wide range of task performance. Specifically, we chose the 30 participants who corresponded to the 30 quantiles of the mean RT of all participants in the data set. Second, we fit the SW-TN and SW-GAM mixtures to the data of the 30 selected participants. To fit the data, we used the Bayesian framework outlined in the last section. For each participant, we collected 4000 samples of each chain after discarding the first 1000 samples as burn-in. Whenever this resulted in inadequate convergence of the chains (i.e., $\hat {R}$ values larger than 1.05), we fit the data again with 1000 additional samples. We continued this process until all $\hat {R}$ values for a given subject were below 1.05. Third, for each participant we determined the mode of the posterior distribution of each model parameter. The modes that were obtained from fitting the SW-TN mixture were used to generate 30 synthetic participants with the SW-TN mixture (i.e., the synthetic SW-TN participants). We used the analogous procedure to obtain 30 synthetic SW-GAM participants, that is, we used the SW-GAM model as data-generating model, and as data-generating values we used the modes that were obtained from fitting the SW-GAM model to the data of the 30 participants selected from the data set of Woods et al., (2015).

After having generated the two synthetic data sets each containing 30 participants each contributing 120 trials, we fit the SW-TN mixture to the 30 synthetic SW-TN participants and the SW-GAM mixture to the 30 synthetic SW-GAM participants. We collected 2000 samples of each chain after discarding the first 1000 samples of each chain as burn-in. Whenever this resulted in a $\hat {R}$ values larger than 1.05, we fit the data again with 1000 additional samples. We continued this process until all $\hat {R}$ values of a given synthetic participant were below 1.05. Finally, we compared whether the modes of the posterior distributions of the synthetic participants correspond to the data-generating values, and we considered the interquartile ranges of the posterior distributions to assess the uncertainty about the parameter values.

In addition, we extended the recovery study in two ways. First, we repeated the recovery study using 1200 trials instead of 120 trials. More precisely, we fit the SW-TN mixture to 30 SW-TN participants each contributing 1200 trials, and analogously for the SW-GAM mixture. Second, we investigated the impact of the drift variance parameter $\sigma ^{2}_{\xi }$. To this aim, we used the four synthetic data sets of the first two recovery studies, but this time the models were fit with drift variance parameter $\sigma ^{2}_{\xi }$ fixed to zero. These models thus assume, falsely, that there is no across-trial variability in the Wald drift rate parameter. Thus, in total we conducted eight model fitting exercises; for each mixture we had two synthetic data sets that differed in whether 120 or 1200 trials were generated, and for each synthetic data set, we fit the full and the restricted model (e.g., drift variance parameter $\sigma ^{2}_{\xi }$ fixed to zero).

### Results

Visual inspection of all chains and confirmation that all parameters had $\hat {R}$ values below 1.05 suggested that the collected samples of all four recovery studies provided a valid approximation to the joint posterior parameter distributions (i.e., adequate convergence of the chains from their starting values to their stationary distributions).

#### Recovery study 1: 30 synthetic participants each contributing 120 trials

**Fig. 2:**
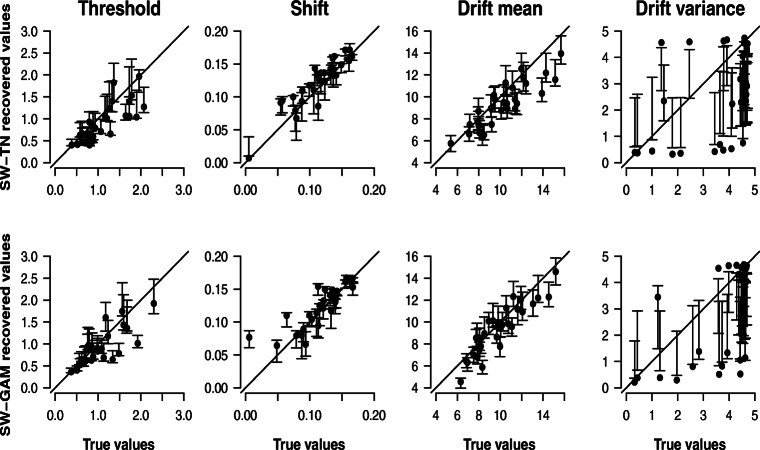
Results for parameter recovery based on 30 synthetic participants each contributing 120 trials. Each panel shows the correlation between the true parameter values (*x*-axis) and the modes of the posterior distributions (*y*-axis) of the SW-TN model (*first row*) and the SW-GAM model (*second row*). *Error bars* and *dots* represent the interquartile range and modes of the posterior distributions, respectively

Figure 2 shows the correlation between the data-generating values (*x*-axis) and the modes of the posterior distributions (*y*-axis) obtained from fitting the SW-TN and SW-GAM mixtures to the data of 30 synthetic SW-TN and SW-GAM participants, respectively, each contributing 120 trials. The error bars represent the interquartile range of the posterior distributions. Under perfect parameter recovery, all dots would lie on the main diagonal, and all error bars would be narrow, indicating little uncertainty about the recovered parameter values. From the figure it is evident that all parameters except for the drift variance parameter can be recovered accurately; overall, there is not much discrepancy between the data-generating values and the modes of the posterior distributions, and only little uncertainty about the true parameter values. However, from Fig. 2 it is also evident that, in the case of the drift variance parameter, many dots strongly deviate from the main diagonal indicating that the drift variance parameter cannot be recovered accurately, and the wide error bars signal high uncertainty. Note that when the posterior distribution is highly skewed, it is possible that the error bars (i.e., the interquartile ranges of the posterior distributions) do not cover the dots (i.e., the modes of the posterior distributions).

**Fig. 3:**
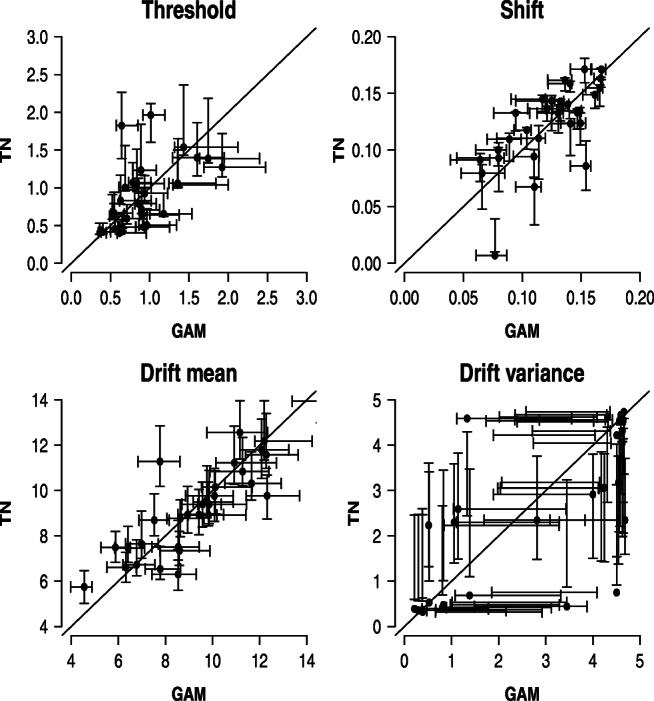
Results for parameter recovery based on 30 synthetic participants each contributing 120 trials. Each panel shows the correlation between the modes of the posterior distribution for a specific parameter of the SW-GAM model (*x*-axis) and the SW-TN model (*y*-axis). *Error bars* represent the interquartile range

To assess whether parameter inferences coincide across the two models, Fig. 3 shows the correlation between the posterior modes of the SW-GAM (*x*-axis) and SW-TN (*y*-axis) model, respectively, together with the error bars representing the interquartile ranges of the posterior distributions. First of all, Fig. 3 suggests that the uncertainty about the true parameter values is comparable in magnitude for both models. Second, it is evident that for all parameters except for the drift variance parameter, the modes of both models are similar since most modes lie close to the main diagonal. However, for the drift variance parameter, the posterior modes of the two models deviate from each other in an unsystematic manner; some are higher estimated by the SW-TN mixture, whereas others are higher estimated by the SW-GAM mixture. Finally, note that Fig. 3 underestimates the true accordance of the two models because the models were fit to different synthetic data sets, that is, the SW-TN and SW-GAM mixture were fit to a data set that was generated with the respective mixture.

#### Recovery study 2: 30 synthetic participants each contributing 1200 trials

**Fig. 4:**
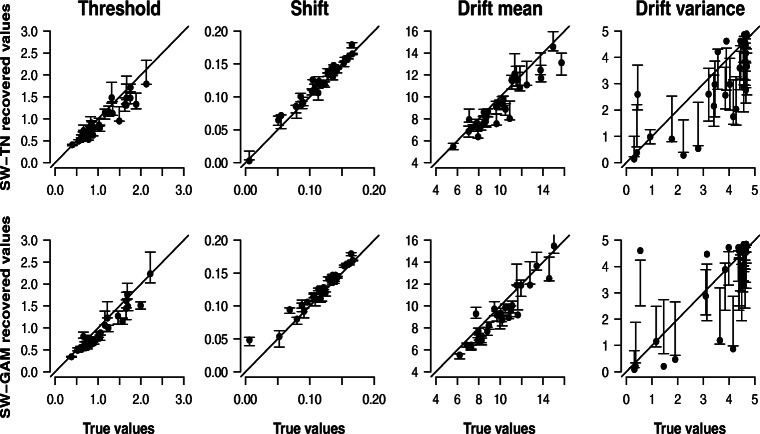
Results for parameter recovery based on 30 synthetic participants each contributing 1200 trials. Each panel shows the correlation between the true parameter values (*x*-axis) and the modes of the posterior distributions (*y*-axis) of the SW-TN model (*first row*) and the SW-GAM model (*second row*). *Error bars* represent the interquartile range

To assess the impact of the number of trials on the parameter recovery, we repeated the same recovery study as presented in the last section, but this time with 1200 trials instead of 120 trials. Figure 4 shows the results. From the figure it is evident that the parameter recovery has improved: The modes of the posterior distribution of the threshold, shift, and drift mean parameter cluster tightly around the main diagonal indicating a close correspondence between the true parameter values and their recovered values. In addition, the narrow error bars signal high certainty about the parameter values. The recovery has also improved in the case of the drift variance parameter, yet systematic deviances and large uncertainty remain. Figure 4 thus suggests that, with a large number of trials, all parameters except for the drift variance parameter can be recovered to a high degree of accuracy.

**Fig. 5:**
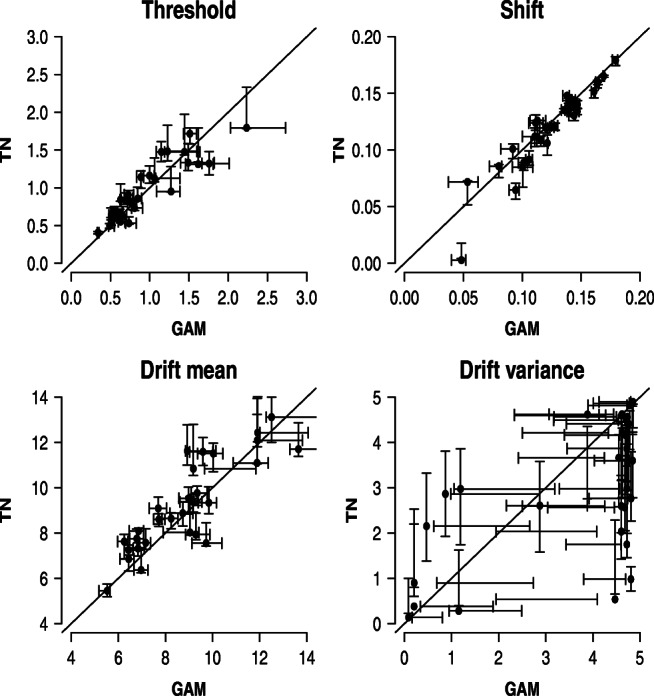
Results for parameter recovery based on 30 synthetic participants each contributing 1200 trials. Each panel shows the correlation between the modes of the posterior distribution for a specific parameter of the SW-GAM model (*x*-axis) and the SW-TN model (*y*-axis). *Error bars* represent the interquartile range

To assess whether parameter inferences coincide across the two models, Fig. 5 shows the correlation between the modes obtained from fitting the SW-GAM (*x*-axis) and SW-TN model (*y*-axis), respectively, together with the error bars representing the interquartile ranges of the posterior distributions. As was the case for the previous recovery study with 120 trials, Fig. 5 suggests that the uncertainty about the true parameter values is comparable in magnitude for both models, and that the parameter inferences for threshold, shift and drift mean are similar for both models. However, in the case of the drift variance parameter, just as when considering 120 trials, the posterior modes of the two models do not match.

#### Recovery study 3: 30 synthetic participants each contributing 120 trials, fixed drift variance parameter

**Fig. 6:**
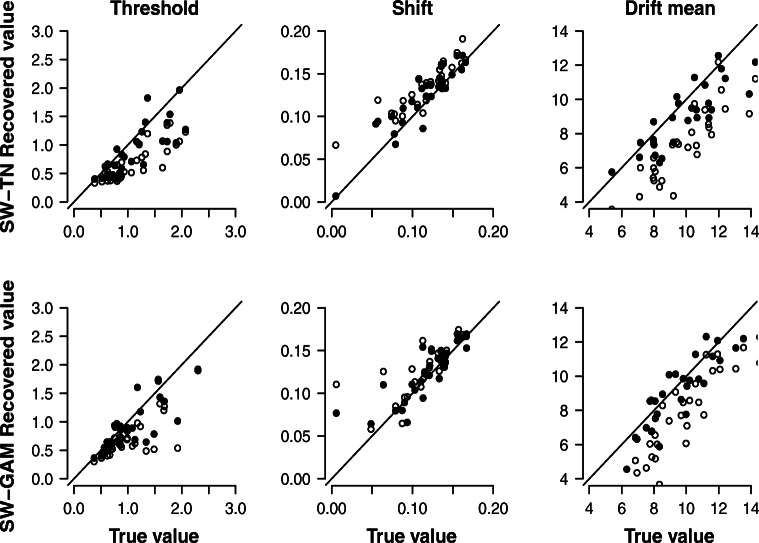
Results for parameter recovery based on 30 synthetic participants each contributing 120 trials. Each panel shows the correlation between the true parameter values (*x*-axis) and the modes of the posterior distribution (*y*-axis) of the SW-TN model (*first row*) and of the SW-GAM model (*second row*). The *filled dots* show the posterior modes of the model with a free drift variance parameter (obtained from recovery study 1), whereas the *unfilled dots* show the posterior modes of the model with a drift variance parameter fixed to zero

In order to investigate the impact of the drift variance parameter on parameter recovery, we used the synthetic data sets from the last two sections, but now the two models were fit with a drift variance parameter fixed to zero (i.e., the vanilla SW model with constant drift). Figure 6 shows the correlation between the data-generating values (x-axis) and the modes of the posterior distributions (y-axis) obtained from fitting the SW-TN and SW-GAM mixture, respectively, to the data of 30 synthetic participants contributing 120 trials. The filled dots show the posterior modes of the model with a free drift variance parameter (obtained from recovery study 1), whereas the unfilled dots show the posterior modes of the model with a drift variance parameter fixed to zero. For a given synthetic participant, the impact of fixing the drift variance parameter to zero can be assessed by choosing a certain value on the x-axis; the difference between the corresponding filled and unfilled dot expresses the disagreement between the two models (i.e., drift variance free to vary vs. drift variance fixed to zero). Figure 6 suggests that, in the case of the threshold and the shift parameter, fixing the drift variance parameter to zero does not harm recovery. However, in the case of the drift mean parameter, fixing the drift variance parameter results in a slight underestimation of the drift mean parameter. Figure 6 also suggests that this pattern is present for both the SW-TN and the SW-GAM mixture.

#### Recovery study 4: 30 synthetic participants each contributing 1,200 trials, fixed drift variance parameter

**Fig. 7:**
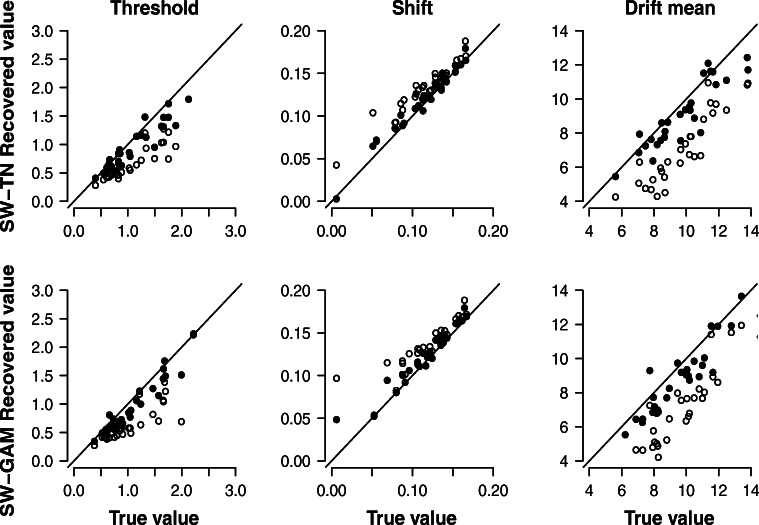
Results for parameter recovery based on 30 synthetic participants each contributing 1200 trials. Each panel shows the correlation between the true parameter values (*x*-axis) and the modes of the posterior distribution (*y*-axis) of the SW-TN model (*first row*) and of the SW-GAM model (*second row*). The *filled dots* show the posterior modes of the model with a free drift variance parameter (obtained from recovery study 2), whereas the *unfilled dots* show the posterior modes of the model with a drift variance parameter fixed to zero

To investigate whether the impact of fixing the drift variance parameter becomes more apparent when using a larger number of RTs, we repeated the recovery study presented in the last section with 1200 trials instead of 120 trials. Figure 7 shows the results. The underestimation of the drift mean parameter when fixing the drift variance parameter is now more systematic. In addition, fixing the drift variance parameter seems to result in a slight underestimation of the threshold and a slight overestimation of the shift parameter. These results suggest that even though the drift variance parameter cannot be recovered accurately (see Figs. 2 and 4), including it to the models does improve recovery of the remaining parameters.

## Application to real data

### Data

We now apply the mixture models to the data set from Woods et al., (2015) kindly provided to us by the authors. This data set consists of 1469 participants, 40.1% men, 10.8% left-handed by self-report, and all between 18 and 65 years. All participants completed a 120-trial simple RT task that required participants to press a response button on a computer gaming mouse as fast as possible once upon detecting a stimulus appearing either in the left or in the right hemifield. As was done by Woods et al., (2015), we excluded RTs less than 110 ms and greater than 1000 ms. More details about the data set and the experiment can be found in Woods et al., (2015; Experiment 1). The data set can be downloaded from the OSF at http://osf.io/av4qn.

### Cognitive modeling analyses

We fit both models (i.e., the SW-TN and SW-GAM mixture) to the data set provided by Woods et al., (2015) using the Bayesian framework outlined above. We collected 4000 samples of each chain after discarding the first 1000 samples of each chain as burn-in. Whenever this resulted in inadequate convergence (i.e., $\hat {R}$ values larger than 1.05), we fit the data again with 1000 additional samples. We continued this process until all $\hat {R}$ values for a given subject were below 1.05.

### Results

**Fig. 8:**
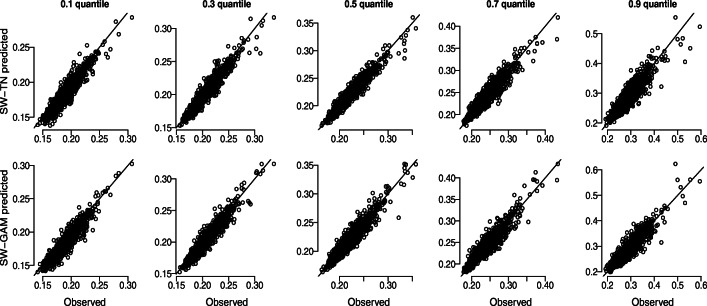
Goodness-of-fit assessment for the SW-TN and SW-GAM mixture in the first and second row, respectively. Five observed and predicted quantiles are compared for the data of Woods et al., (2015; *n* = 1469)

Visual inspection of the chains and confirmation that all parameters had $\hat {R}$ values below 1.05 suggested that the collected samples provided a valid approximation to the joint posterior parameter distribution. To assess each model’s goodness-of-fit, we compare for each participant the observed .1, .3, .5, .7., .9 quantiles to the predicted quantiles (see also Ratcliff and Van Dongen (2011)).^3^ The predicted quantiles were obtained by generating 120 RTs using the modes of the posterior distributions, for both mixtures separately. Figure 8 suggests that both mixtures provide a good account of the data as the predicted quantiles closely correspond to the observed quantiles.

**Fig. 9:**
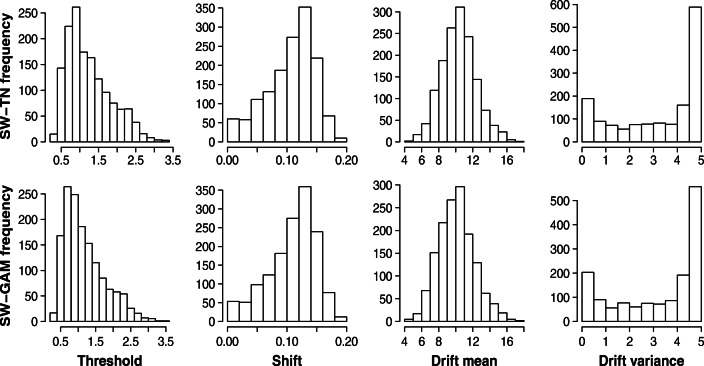
Histograms for the modes of the posterior distributions obtained from fitting the SW-TN model to the data of Woods et al., (2015; *n* = 1469)

Figure 9 shows the histograms for the modes of the posterior distributions of all participants for the SW-TN and SW-GAM mixture. First of all, it is evident that the histograms look very similar for both mixtures. Second, the mode of the threshold parameter is around 1, the mode of the shift parameter around .13, and the mode of the drift mean parameter is around 10. Third, the mode of the drift variance parameter has a bimodal distribution and should not be interpreted due to poor parameter recovery and high uncertainty.

**Fig. 10:**
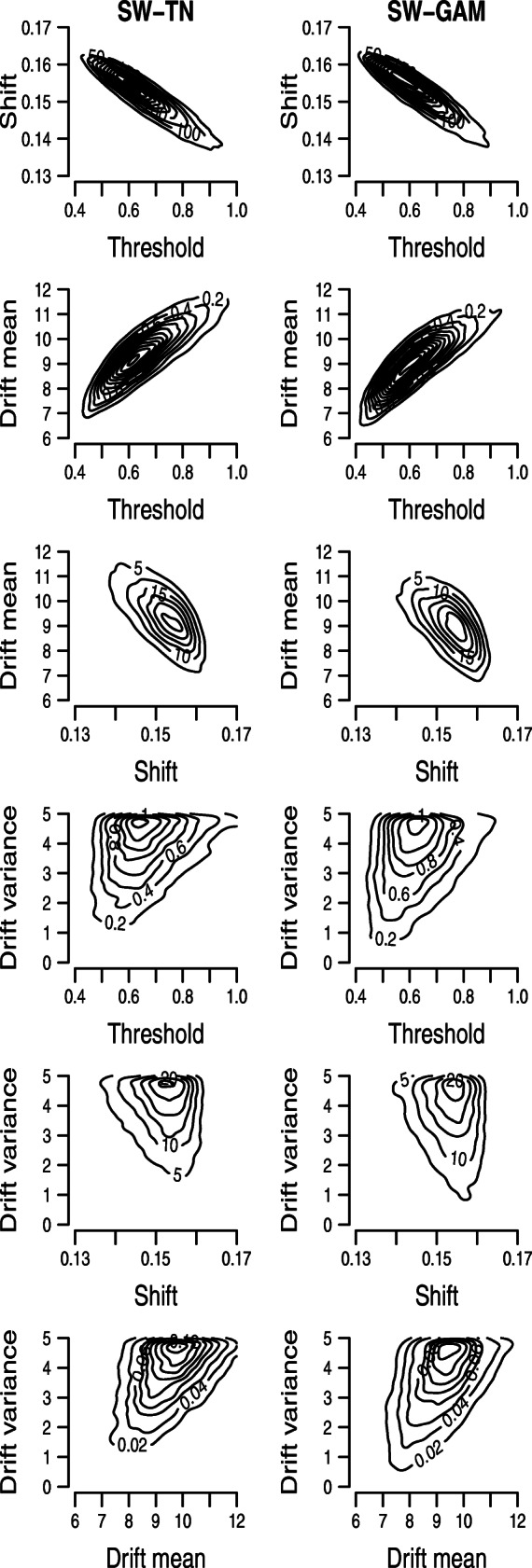
Contour plots represent the correlation between the parameters of the SW-mixture models based on the posterior samples of a representative participant from the Woods et al., data set (2015; *n* = 1469). *Left column*: SW-TN model. *Right column*: SW-GAM model

**Fig. 12:**
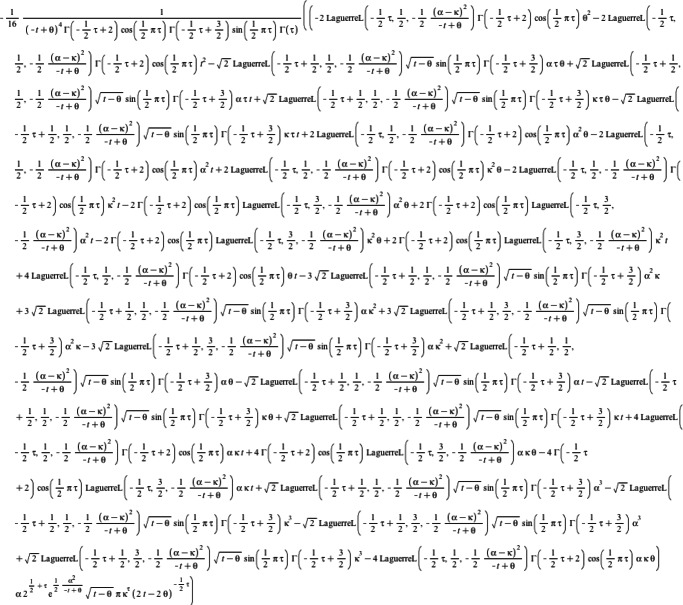
Solution of $ {\int \limits }_{0}^{\infty } \text {SW}(t ; \alpha , \ \theta , \ \xi ) \cdot \text {GAM}(\xi ; \ \text {shape} = \kappa , \text {rate} = \tau ) \ \mathrm {d}{\xi }$, with *κ* the shape parameter and *τ* the rate parameter, derived by Maple

Figure 10 shows contour plots that represent the correlation between the parameters of the SW-TN and SW-GAM mixture for a representative participant.^4^ A comparison between the left and right columns suggests that the correlation pattern is consistent across both mixtures. Second, there is no substantial correlation between drift variance and the remaining parameters (bottom three rows of Fig. 10)—a foreseeable finding given the large uncertainty about the drift variance parameter observed in the recovery studies. Third, the top row of Fig. 10 shows a strong negative correlation between the threshold and shift parameter suggesting that a higher threshold can be compensated by lower shift. Fourth, the second row of Fig. 10 shows a strong positive correlation between the threshold and drift mean parameter suggesting that a higher threshold can be compensated by a higher drift mean. Finally, a–at first glance—counterintuitive correlation is shown in the third row: The shift parameter is negatively correlated with the drift mean parameter. This finding is driven by the correlations shown in the first two rows of Fig. 10. It is evident that increasing the shift parameter results in a lower threshold (first row), and a lower threshold results in a lower drift mean (second row).

## Discussion

This article illustrated how the shifted-Wald model can be extended to incorporate across-trial variability in the Wald drift rate parameter. More specifically, we assumed that the trial-dependent drift rate is governed by either a truncated normal distribution or a gamma distribution. We showed that analytical solutions exist for the resulting distributions of the first-arrival times, and explicitly derived them. In addition, we explained how they can be implemented in a Bayesian framework. Due to the complexity of the analytical solution for the SW-GAM mixture, we only implemented the analytical solution for the SW-TN mixture, but provided a sampling-based implementation for the SW-GAM mixture. We confirmed the adequacy of both implementations in four recovery studies, and demonstrated their practicality on the example of a data set comprised of 1469 participants (Woods et al., 2015).

Our recovery studies showed that (1) all parameters except for the drift variability parameter can be recovered accurately; (2) parameter recovery of the drift variability parameter is problematic, yet including this parameter results in more accurate parameter recovery of the remaining model parameters; and (3) parameter inferences are consistent across both the SW-TN and the SW-GAM mixtures. Our application to real data also suggests that the results are consistent across both mixtures. In addition, we showed that both mixtures provide a good account of the data, but that the shift, threshold, and drift mean parameters are correlated.

Our models can be readily applied to a large variety of one-choice RT tasks—tasks that require participants to “initiate a simple, preprogrammed response to a simple triggering signal” (p. 49; Luce (1986)). Examples of such tasks are simple RT tasks, go/no-go tasks, and temporal-cueing tasks (Heathcote, 2004; Jepma et al., 2012; Kamienkowski et al., 2011; Luce, 1986; Schwarz, 2001; Smith, 2000). Next to one-choice RT tasks, our models can also be applied to tasks from other domains involving duration phenomena. Examples are job completion times of employees in economics (Desmond and Chapman, 1993), and organ transit time distributions of vascular markers (so called washout curves) in biology (Weiss, 2012). In addition, our implementations can be extended to account for contaminant processes by assuming that the observed RT originates from two processes—the Wiener diffusion process toward a single absorbing boundary and a contaminant process that can be modeled as a uniform distribution between a lower and upper RT bound (Jepma et al., 2012; Ratcliff & Tuerlinckx, 2002; Zeigenfuse & Lee, 2010). Yet another possible extension of our implementations is to also account for commonalities and differences across participants using a Bayesian hierarchical framework (Anders et al., in press; Jepma et al., 2012; Shiffrin et al., 2008).

Our results suggest that including variability in the Wald drift rate parameter may be worthwhile since it improves the parameter recovery of the remaining model parameters (e.g., Burbeck & Luce, 1982; Ratcliff & Strayer, 2014; Ratcliff & Tuerlinckx, 2002; Ratcliff & Van Dongen, 2011; Ratcliff & Van Zandt, 1999; for an alternative perspective see van Ravenzwaaij et al., (2017)). A crucial advantage of our implementation is that it avoids having to fit the data by simulation (e.g., Ratcliff and Van Dongen, 2011; Ratcliff & Strayer, 2014). In addition, our results show that the parameter inferences are not affected by whether we use the analytical solution for the SW-TN mixture or the sampling-based solution for the SW-GAM mixture. This suggests that the inference is robust to the type of distribution on the drift rate parameter and to the type of implementation.

Our recommendation to include across-trial variability in the Wald drift rate parameter relied on a recovery study with generated data from a shifted-Wald model that incorporates across-trial variability. In real data, across-trial variability in the drift rate may be absent or negligible. In order to assess the extent to which the data support the inclusion of the across-trial variability in the drift rate, we recommend researchers to use the Bayes factor (Etz & Wagenmakers, 2017; Jeffreys, 1961; Vandekerckhove et al., 2015); this can be accomplished using bridge sampling and the data pool reported in this article (Gronau et al., in press). As a side note, even though it would theoretically be possible to add across-trial variability in the shift parameter and the threshold parameter as well, we believe that for realistic sample sizes the Bayes factor will indicate that the model without theses variabilities shows better predictive performance. Here we focus on drift rate variability because it is the most consequential and the most interesting from a psychological perspective (see also Ratcliff, 1978).

Finally, our results can be used to create informative prior distributions for the SW-TN and SW-GAM mixtures. Since we fit both mixtures to a data set comprised of 1469 participants, the histograms for the posterior modes (Fig. 9) offer a good description of which parameter values can be expected when modeling one-choice RT data. One way to create informative prior distributions based on Fig. 9 is to use uniform distributions with a lower and upper bound corresponding to the bounds shown in that figure (see Matzke & Wagenmakers, 2009). Even more informative prior distributions can be used by fitting a positive-valued distribution to the distributions of the modes, and then using the resulting distributions as prior distributions.^5^

To conclude, we have extended the SW model to incorporate across-trial variability in the Wald drift rate parameter, and derived distributions of the first-arrival times under the assumption that individual-trial drift rates are governed either by a truncated normal distribution or a gamma distribution. Our extended model can be applied to a broad range of data sets involving duration phenomena and can easily be extended to account for contaminant processes and hierarchical structure.

## Figures and Tables

**Fig. 11:**
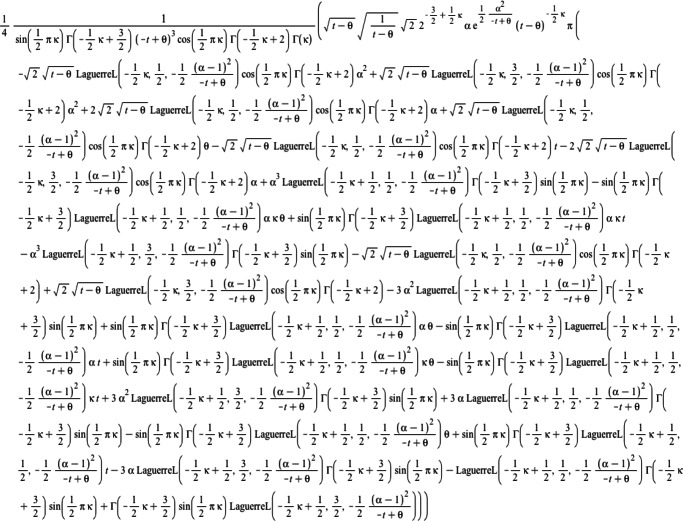
Solution of $ {\int \limits }_{0}^{\infty } \text {SW}(t ; \alpha , \ \theta , \ \xi ) \cdot \text {GAM}(\xi ; \ \kappa , \tau = 1) \ \mathrm {d}{\xi }$, with *κ* the shape parameter and *τ* the rate parameter, derived by Maple

## Appendix

### Analytical solution of the SW-GAM mixture

The analytical solution for the SW-GAM mixture was obtained by assuming that on each trial, RT follows a SW distribution that depends in part on a trial-dependent drift rate parameter; the drift rate parameter itself is modelled as a draw from a gamma distribution, and this parameter is subsequently integrated out. Table 1 and Figs. 11 and 12 show the results. The results are presented for several scenarios that differ in the number of parameters fixed to one. To obtain the results we used standard integration techniques, but also the computer software Maple (2015). We also tried to derive the results using Mathematica (Wolfram Research, 2010). The results were either identical to the ones provided by Maple, or else Mathematica failed to provide any results.

**Table 1:** Analytical solutions for the first-arrival times (third column) assuming a SW distribution on RT (first column) with a drift rate *ξ* that varies according to a Gamma distribution parameterized by the shape parameter *κ* and the rate parameter *τ* (second column)

RT distribution with	Drift rate distribution	RT Distribution with
trial-independent drift		trial-dependent drift
SW(*t*; *α*, *𝜃*, *ξ*)	GAM(*ξ*; *κ*, *τ*)	$ f(t; \ \alpha , \ \theta , \ \kappa , \ \tau ) = {\int \limits }_{0}^{\infty } \text {SW}\cdot \text {GAM} \ \mathrm {d}{\xi }$
SW(*t*; *α* = 1, *𝜃*, *ξ*)	GAM(*ξ*; *κ* = *τ* = 1)	$\frac {{\exp \left ({- \frac {1}{2 (t - \theta ) }} \right ) }}{2 (t - \theta )^{2}}$
	GAM(*ξ*; *κ* = 1,*τ*)	$ \frac {\tau }{2 (t - \theta )^{2} } \cdot {{\exp \left ({\frac {\tau (\tau - 2) }{2(t - \theta )}} \right )} \cdot \left (1+ {\text {erf}\left ({\frac {1 - \tau }{ \sqrt {2(t - \theta )}}}\right )} \right )}$
	GAM(*ξ*; *κ*, *τ* = 1)	$\sqrt { \frac {2^{\kappa - 3}}{\pi (t - \theta )^ 3 }} \cdot \frac {\gamma (\frac {1}{2} \kappa )}{\gamma (\kappa )} \cdot \exp \left (- \frac {1}{2 (t - \theta ) }\right ) $
SW(*t*; *α*, *𝜃*, *ξ*)	GAM(*ξ*; *κ* = 1,*τ*)	$ \frac {\alpha \tau }{2 {(t - \theta )}^{2} } \cdot { {\exp \left ({- {\frac {(2 \alpha - \tau ) \tau }{2 (t - \theta ) } }} \right ) } \cdot \left (1 + {\text {erf}\left ({\frac {\alpha - \tau }{\sqrt {2 (t - \theta )}}}\right )} \right )} $
	GAM(*ξ*; *κ*, *τ* = 1)	See Fig. 11
	GAM(*ξ*; *κ*, *τ*)	See Fig. 12

The analytical solutions were derived with standard integration techniques and Maple. Each row differs depending on which parameter of the SW and gamma distribution were fixed to one

From the table and the figures it is evident that assuming a decision threshold *α* fixed to one results in relatively short solutions that are straightforward to implement and computationally inexpensive; however, letting decision threshold *α* free to vary results in lengthy solutions involving the Laguerre polynomial (https://www.maplesoft.com/support/help/maple/view.aspx?path=LaguerreL; retrieved 2016, July 14; chapter 3 in Bayin, 2006; Koepf, 1997). Note that LaguerreL(n, a, x) is a function implemented in Maple that computes the n^th^ Laguerre polynomial.

In principle, it is possible to add the solution displayed in Fig. 12—the solution where all relevant parameters are free to vary—to JAGS following the procedure described by Wabersich and Vandekerckhove (2013). Since the LaguerreL function is not contained in JAGS, this would involve programming the LaguerreL function in C++, a programming language that is used for the JAGS implementation. In order to program the Laguerre polynomial one can use its definition as the product of a generalized binomial coefficient and the Kummer confluent hypergeometric function of the first kind (also known as Kummer’s function of the first kind; Equation 10 in Georgiev & Georgieva-Grosse, 2010):

5 $$  \text{LaguerreL}(n, a, x) = {L^{a}_{n}}(x) = \binom{n+a}{n} \cdot \text{KummerM}(-n, a+1, x). $$

The binomial coefficient in Eq. 5 can be rewritten using:

6 $$  \binom{n+a}{n} = \frac{\Gamma{ \left (n + a + 1 \right ) }} {\Gamma \left( n + 1 \right) \cdot {\Gamma} \left (a + 1 \right )} \ . $$

The Kummer confluent hypergeometric function of the first kind is already implemented in C++. Note that the analytical solutions presented in Figs. 11 and 12 involve Laguerre functions with negative inputs, resulting in negative inputs for the gamma function in Eq. 6. Negative inputs to the gamma function are not allowed in C++ that is used for the JAGS implementation. One therefore needs to use the following definition of the gamma function for negative inputs excluding integers (otherwise the sine is zero):

7 $$  {\Gamma}(x)=\frac{\pi}{x {\Gamma}(-x) \sin(- \pi x)} \ , \ x \in \mathbb{R}^{-}\setminus \mathbb{Z}^{-}_{0} $$

(http://algolist.manual.ru/maths/count_fast/gamma_function.php; retrieved July 14, 2016). The classical identities Γ(*x*) = (*x* − 1)! and ${\Gamma }(x)=\frac {\Gamma (x+1)}{x} $ suggest that the gamma function is not defined for negative integers including zero (i.e., $x \notin \mathbb {Z}^{-}_{0}$). However, Fisher and Kılıcman (2012) proved that the gamma function can be defined for negative integers as follows:

8 $$  {\Gamma}(x)= \frac{ (-1)^{-x} }{(- x) !} \ \left (\mathrm{g} (- x) - \gamma \right ) \ , \ x \in \mathbb{Z}^{-}, $$

with $\mathrm {g} (- x) = {\sum }_{i=1}^{-x} \frac {1}{i} $ and $\gamma = - {\Gamma }(0) = \lim _{n \to \infty } {\sum }_{k=1}^{n} \left (\frac {1}{k} - \ln (n) \right )$ which is Euler’s constant. Thus, Γ(0) = −*γ*. This information, together with Eqs. 5–8, is sufficient to implement the Laguerre polynomial in C++ and R.

### JAGS model files

This section presents the JAGS model files to implement the SW-TN and SW-GAM mixture. All relevant code can be downloaded from GitHub at https://github.com/HelenSteingroever/jags-wald/releases.

The JAGS model code for the SW-TN mixture is as follows:

The JAGS model code for the SW-GAM mixture:

Note that the T(0,) argument ensures positive values and that the second argument of the normal distribution refers to the precision being defined as the reciprocal of the variance (i.e., *λ* = 1/*σ*^2^). In addition to the dunif(0, 5) prior (i.e., a uniform prior ranging from 0 to 5, that is, $\mathcal {U}(0, 5)$) on the drift variance, we also tried a dunif(0, 2) prior for our recovery studies. The problem with this parameter is that, in the recovery studies, a lot of mass of many posterior distributions often lies close to the upper bound even though the true values did not—a finding that did not affect the remaining parameter inferences, but that we observed independently of whether an upper bound of 2 or 5 was used. We assume that this pattern will remain even if we use a yet higher upper limit (i.e., the principle of stable estimation; Edwards et al., 1963). We therefore decided to use a dunif(0, 5) prior on the drift variance for all analyses presented in this article.

## Footnotes

1 A more complete derivation is available on the Open Science Framework (OSF; http://osf.io/av4qn).

2 Note that the appendix presents several analytical solutions that differ depending on which parameters of the SW and gamma distribution are set to fixed values.

3 Note that we did not compare each model’s performance to the performance of a benchmark model (e.g., a model without random drift) because our purpose is parameter estimation and not model selection.

4 The correlations for all remaining participants can be examined using the data and R code provided on the OSF at http://osf.io/av4qn. This also involves fitting the model using the R code provided on GitHub at https://github.com/HelenSteingroever/jags-wald/releases.

5 The modes can be downloaded from the OSF at http://osf.io/av4qn.

## References

[CR1] Anders, R., Alario, F.-X., & Van Maanen, L. (in press). The shifted-Wald distribution for response time data analysis. *Psychological Methods*. 10.1037/met0000066 26867155

[CR2] Banerjee AK, Bhattacharyya G (1979). Bayesian results for the inverse Gaussian distribution with an application. Technometrics.

[CR3] Bayin S (2006). Mathematical methods in science and engineering.

[CR4] Betro B, Rotondi R (1991). On Bayesian inference for the inverse Gaussian distribution. Statistics & Probability Letters.

[CR5] Burbeck SL, Luce RD (1982). Evidence from auditory simple reaction times for both change and level detectors. Perception & Psychophysics.

[CR6] Desmond A, Chapman G (1993). Modelling task completion data with inverse Gaussian mixtures. Applied Statistics.

[CR7] Desmond A, Yang Z (2011). Score tests for inverse Gaussian mixtures. Applied Stochastic Models in Business and Industry.

[CR8] Donkin C, Brown SD, Heathcote A (2009). The overconstraint of response time models: Rethinking the scaling problem. Psychonomic Bulletin & Review.

[CR9] Edwards W, Lindman H, Savage LJ (1963). Bayesian statistical inference for psychological research. Psychological Review.

[CR10] Etz A, Wagenmakers E-J (2017). JBS Haldane’s contribution to the Bayes factor hypothesis test. Statistical Science.

[CR11] Fisher B, Kılıcman A (2012). Some results on the gamma function for negative integers. Applied Mathematics & Information Sciences.

[CR12] Forstmann B, Ratcliff R, Wagenmakers E-J (2016). Sequential sampling models in cognitive neuroscience: Advantages, applications, and extensions. Psychology.

[CR13] Gelman A, Rubin D (1992). Inference from iterative simulation using multiple sequences. Statistical Science.

[CR14] Georgiev, G. N., & Georgieva-Grosse, M. N. (2010). An application of the zeros of Laguerre polynomials. In *International conference on electromagnetics in advanced applications (ICEAA)* (pp. 637–640).

[CR15] Gronau, Q. F., Sarafoglou, A., Matzke, D., Ly, A., Boehm, U., Marsman, M., & et al. (in press). A tutorial on bridge sampling. *Journal of Mathematical Psychology*. 10.1016/j.jmp.2017.09.005 PMC5699790 29200501

[CR16] Heathcote A (2004). Fitting Wald and ex-Wald distributions to response time data: An example using functions for the s-PLUS package. Behavior Research Methods, Instruments, & Computers.

[CR17] Heathcote, A., Brown, S. D., & Wagenmakers, E.-J. (2015). An introduction to good practices in cognitive modeling. In *An introduction to model-based cognitive neuroscience* (pp. 25–48): Springer.

[CR18] Jeffreys H (1961). Theory of probability.

[CR19] Jepma M, Wagenmakers E-J, Nieuwenhuis S (2012). Temporal expectation and information processing: A model-based analysis. Cognition.

[CR20] Kamienkowski JE, Pashler H, Dehaene S, Sigman M (2011). Effects of practice on task architecture: Combined evidence from interference experiments and random-walk models of decision making. Cognition.

[CR21] Koepf W (1997). Identities for families of orthogonal polynomials and special functions. Integral Transforms and Special Functions.

[CR22] Logan GD, Van Zandt T, Verbruggen F, Wagenmakers E-J (2014). On the ability to inhibit thought and action: General and special theories of an act of control. Psychological Review.

[CR23] Luce, R. D. (1986). *Response times* (No. 8). Oxford University Press.

[CR24] Maple (2015). *Maplesoft a division of Waterloo Maple Inc., Waterloo, Ontario*.

[CR25] Matzke D, Wagenmakers E-J (2009). Psychological interpretation of the ex-Gaussian and shifted Wald parameters: A diffusion model analysis. Psychonomic Bulletin & Review.

[CR26] Plummer, M. (2003). JAGS: A program for analysis of Bayesian graphical models using Gibbs sampling.

[CR27] Ratcliff R (1978). A theory of memory retrieval. Psychological Review.

[CR28] Ratcliff R, Smith PL, Brown SD, McKoon G (2016). Diffusion decision model: Current issues and history. Trends in Cognitive Sciences.

[CR29] Ratcliff R, Strayer D (2014). Modeling simple driving tasks with a one-boundary diffusion model. Psychonomic Bulletin & Review.

[CR30] Ratcliff R, Tuerlinckx F (2002). Estimating parameters of the diffusion model: Approaches to dealing with contaminant reaction times and parameter variability. Psychonomic Bulletin & Review.

[CR31] Ratcliff R, Van Dongen HP (2011). Diffusion model for one-choice reaction-time tasks and the cognitive effects of sleep deprivation. Proceedings of the National Academy of Sciences.

[CR32] Ratcliff R, Van Zandt T (1999). Connectionists and diffusion models of reaction time. Psychological Review.

[CR33] Robert CP (1995). Simulation of truncated normal variables. Statistics and Computing.

[CR34] Schwarz W (2001). The ex-Wald distribution as a descriptive model of response times. Behavior Research Methods, Instruments, & Computers.

[CR35] Shiffrin RM, Lee MD, Kim W, Wagenmakers E-J (2008). A survey of model evaluation approaches with a tutorial on hierarchical Bayesian methods. Cognitive Science.

[CR36] Smith PL (2000). Stochastic dynamic models of response time and accuracy: A foundational primer. Journal of Mathematical Psychology.

[CR37] Terry A, Marley A, Barnwal A, Wagenmakers E-J, Heathcote A, Brown SD (2015). Generalising the drift rate distribution for linear ballistic accumulators. Journal of Mathematical Psychology.

[CR38] Vandekerckhove, J., Matzke, D., & Wagenmakers, E.-J. (2015). Model comparison and the principle of parsimony. In J. Busemeyer, J. Townsend, Z. J. Wang, & A. Eidels (Eds.) *Oxford handbook of computational and mathematical psychology*. Oxford: Oxford University Press.

[CR39] van Ravenzwaaij D, Donkin C, Vandekerckhove J (2017). The EZ diffusion model provides a powerful test of simple empirical effects. Psychonomic Bulletin & Review.

[CR40] Wabersich, D., & Vandekerckhove, J. (2013). Extending JAGS: A tutorial on adding custom distributions to JAGS (with a diffusion model example). *Behavior Research Methods*, 1–14. 10.3758/s13428-013-0369-3 23959766

[CR41] Wald A (1947). Sequential analysis.

[CR42] Weiss M (2012). A model for transit time distributions through organs that accounts for fractal heterogeneity. Journal of Theoretical Biology.

[CR43] Whitmore, G. (1986). Normal-gamma mixtures of inverse Gaussian distributions. *Scandinavian Journal of Statistics*, 211–220.

[CR44] Wolfram Research Inc (2010). Mathematica 8.0.

[CR45] Woods DL, Wyma JM, Yund EW, Herron TJ, Reed B (2015). Factors influencing the latency of simple reaction time. Frontiers in Human Neuroscience.

[CR46] Zeigenfuse MD, Lee MD (2010). A general latent assignment approach for modeling psychological contaminants. Journal of Mathematical Psychology.

